# Chloroplast digestion and the development of functional kleptoplasty in juvenile *Elysia timida* (Risso, 1818) as compared to short-term and non-chloroplast-retaining sacoglossan slugs

**DOI:** 10.1371/journal.pone.0182910

**Published:** 2017-10-11

**Authors:** Elise Marie Jerschabek Laetz, Heike Wägele

**Affiliations:** 1 Center for Molecular Biodiversity Research (ZMB), Zoological Research Museum Alexander Koenig Adenauerallee 160 Bonn, Germany; 2 Institute for Evolutionary Biology and Ecology, University of Bonn, An der Immenburg 1 Bonn, Germany; College of Charleston, UNITED STATES

## Abstract

Sacoglossan sea slugs are the only metazoans known to perform functional kleptoplasty, the sequestration and retention of functional chloroplasts within their digestive gland cells. Remarkably, a few species with this ability can survive starvation periods of 3–12 months likely due to their stolen chloroplasts. There are no reports of kleptoplast transfer from mother slug to either eggs or juveniles, demonstrating that each animal must independently acquire its kleptoplasts and develop the ability to maintain them within its digestive gland. We present here an investigation into the development of functional kleptoplasty in a long-term kleptoplast retaining species, *Elysia timida*. Laboratory-reared juvenile slugs of different post-metamorphic ages were placed in starvation and compared to 5 known short-term retaining slug species and 5 non-retaining slug species. The subsequent results indicate that functional kleptoplasty is not performed by *E*. *timida* until after 15 days post-metamorphosis and that by 25 days, these animals outlive many of the short-term retention species. Digestive activity was also monitored using lysosomal abundance as an indicator, revealing different patterns in starving juveniles versus adults. Starved juveniles were reintroduced to food to determine any differences in digestive activity when starvation ends, resulting in an increase in the number of kleptoplasts, but no overall change in lysosomal activity. By revealing some of the changes that occur during early development in these animals, which begin as non-kleptoplast-retaining and grow into long-term retaining slugs, this investigation provides a basis for future inquiries into the origin and development of this remarkable ability.

## Introduction

The heterobranch (Gastropoda) clade Sacoglossa is well known for some member’s ability to steal functional chloroplasts from their algal food, incorporate them and stave off death by starvation when new food sources are unavailable [1–3]. This phenomenon, termed functional kleptoplasty [4] due to the chloroplasts continued fixation of carbon, occurs in multiple sacoglossan lineages although only six species are confirmed as long-term (two to ten months) retention (LtR) [5–8]. Numerous other species are capable of varying degrees of kleptoplasty ranging from those capable of short-term (two to eight weeks) retention (StR), to those that don’t retain kleptoplasts (NR) [5]. Most sacoglossan species have not been investigated regarding kleptoplast retention.

Much of the recent research on the evolution of kleptoplasty has centered on *Elysia timida* (Risso, 1818) [9–19], an LtR species living in shallow Mediterranean waters [5,9,20–22]. Most evidence shows *El*. *timida* to be a stenophagous species, feeding only on the chlorophyte *Acetabularia acetabulum* Linnaeus, 1758 which is abundantly found in large meadows across the sublittoral zone [9,23–28], although other reports suggest it may have another food source [12,21,22]. A secondary food source has not been identified, however by barcoding or any other distinctive identification method [23]. *A*. *acetabulum*’s bi-phasic life cycle leaves barely any food for *El*. *timida* during the late summer and early autumn months when the algae enter a generative phase consisting of microscopic, planktonic gametes [21]. This lack of available food may have driven *El*. *timida* to develop long-term retention. Whether or not these slugs benefit from the photosynthates produced by their enslaved chloroplasts is debated [29–33] although photosynthates are produced and they may be what allows a slug to survive extended starvation periods [17,34–36]. Thus, food availability may have shaped the *El*. *timida* life cycle [21,25].

Juvenile *El*. *timida* slugs hatch as either veliger larvae or shell-less juveniles depending on food availability and temperature [9,21,37,38]. Regardless of the developmental stage in which they hatch, there are no reports of kleptoplasts within their tissues before they begin to feed, showing that kleptoplasts are not inherited from the parent slugs, rather they must be newly acquired by each new generation [9,24,39,40]. Young *El*. *timida* are assumed incapable of long-term kleptoplast retention, directly digesting chloroplasts for a few weeks until functional kleptoplasty can develop, however little evidence exists to substantiate this claim. Marín and Ros [1993] state that kleptoplasts are retained in the digestive gland of 12 Days Post-Metamorphosis (DPM) juveniles, although a detailed description of how this was determined is missing. A juvenile non-retention period has been demonstrated in *Elysia chlorotica* Gould, 1870, a LtR form that feeds on the heterokontophyte *Vaucheria litorea* Agardh, 1823 [41,42], however most sacoglossans feed on chlorophytes so *El*. *chlorotica* may not be the best model species to represent the entire group.

Recent studies have provided evidence suggesting kleptoplasts from different algal species have varying fitness levels, meaning the algal species is also important to the development of functional kleptoplasty [14,23,43,44]. *Elysia viridis* (Montagu, 1804) is either classified as a LtR or a StR, with its longevity in starvation and fitness depending on the algal species ingested [6,45]. Juvenile *El*. *timida* feed on the same algae as adult *El*. *timida*, where the kleptoplasts are known to be robust (functional and not showing signs of degradation for up to 2 months). This means any difference between adult *El*. *timida* and juvenile *El*. *timida* likely stems from a difference in the slug’s digestive system and not algal or chloroplast type. Little is known about juvenile longevities in starvation and whether *El*. *timida* displays a transient kleptoplasty phase as observed in *El*. *chlorotica*, defined by Pelletreau et al. (2012) as the transitional stage between non-retention and long-term kleptoplast retention [42]. Laetz et al. (2016) measured functional kleptoplast and lysosome abundances in starving adults, finding an inverse relationship. The increase in lysosomes within the digestive gland—but not outside in other tissues—as the number of functional kleptoplasts decrease, suggests that these animals are digesting their incorporated kleptoplasts.

This study assesses whether the functional kleptoplast decrease and presumed digestion observed in adults [46] is the same in juveniles, if ingested chloroplasts (cps) are actually digested in juveniles, and when exactly juvenile slugs gain the ability to maintain cps in their tissues. Chlorophyll *a* autofluorescence is used as an indicator of functional chloroplast presence. Individual juveniles of different ages were measured every day during starvation to examine the digestion of functional kleptoplasts within their digestive glands and uncover when functional kleptoplasty is established. Longevity tests were conducted on both, the animals used in testing and in unhandled animals (animals not subjected to staining and microscopy), to determine if handling stress affected the outcomes. Lysosomal activity, as an indicator of functional digestion, was also monitored throughout various starvation points to determine if chloroplast decrease and lysosomal abundance display the same patterns in juveniles as observed in adults [46]. The StR species *Thuridilla hopei* (Vérany, 1853) and LtR/StR *Elysia viridis* were assessed in Laetz et al. (2016), showing different kleptoplast digestive patterns than those seen in LtR form *Elysia timida*. This study also presents starvation data on five StR forms, *Elysia patina* Ev. Marcus, 1980, *Elysia papillosa* Verrill, 1901, *Elysia cornigera* Nuttall, 1989, *Bosellia mimetica* Trinchese, 1891 and *Thuridilla hopei*, as well as five NR forms, *Placida dendritica* (Alder & Hancock, 1843), *Elysia tuca* Ev. Marcus & Er. Marcus, 1967, *Elysia marcusi* (Ev. Marcus, 1972), *Ercolania fuscata* (Gould, 1870) and *Ercolania viridis* (A. Costa, 1866) as a comparison to juvenile *Elysia timida*. Some of these species were measured for photosynthetic activity (PA) and / or starvation longevity (SL) in previous reports (PA: *Er*. *fuscata*, *Er*. *viridis;* PE+SL: *El*. *patina*, *El*. *papillosa*, *El*. *tuca*, *El*. *marcusi*, *El*. *cornigera;* SL: *P*. *dendritica* [5,47,48]) and the results found there are compared to those presented here, whereas other species are reported here for the first time (SL: *Er*. *fuscata*, *Er*. *viridis;* PA: *Placida dendritica;* PA+SL: *T*. *hopei* and *B*. *mimetica*).

## Material and methods

**Fig 1:**
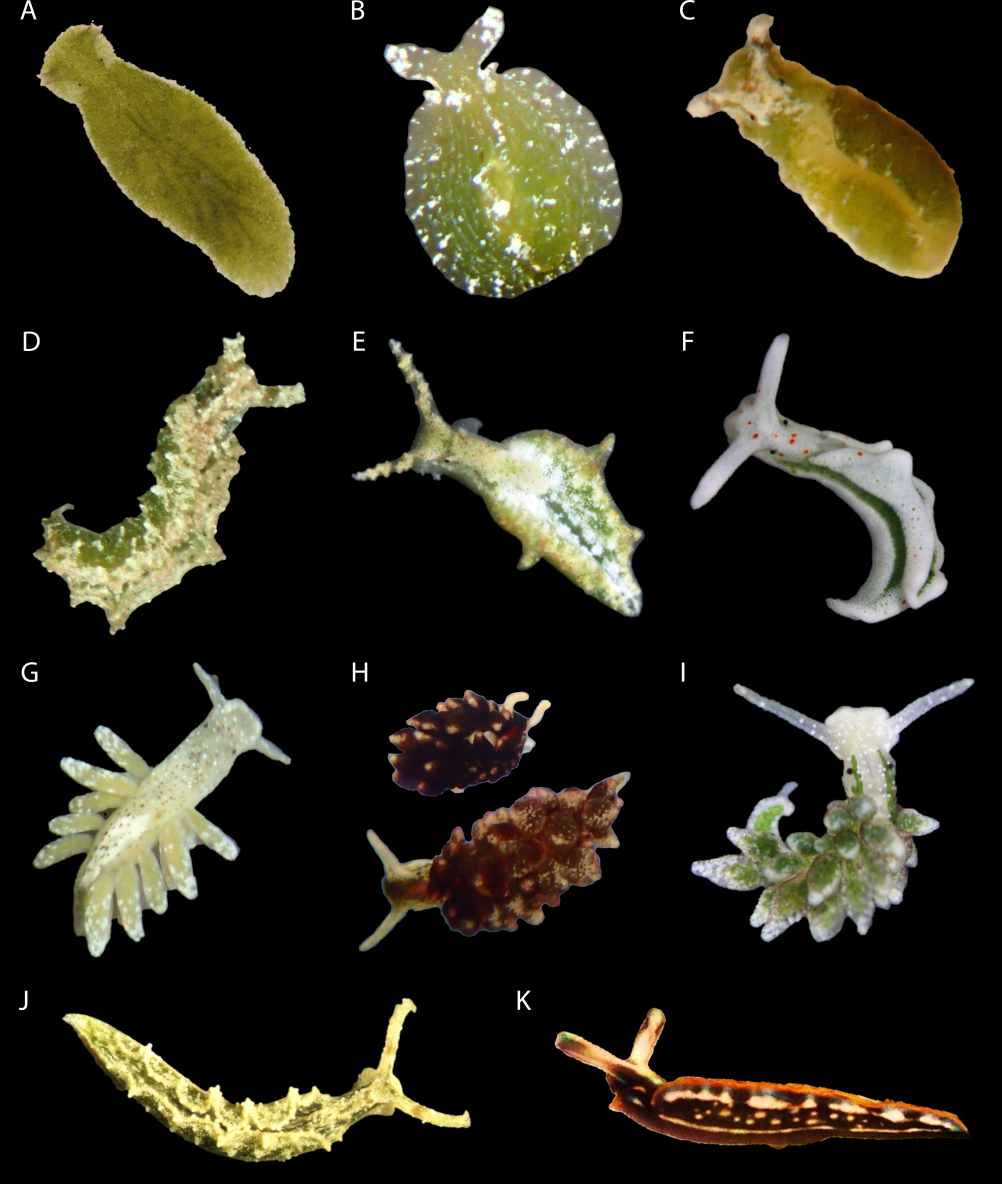
Species investigated. A–*Bosellia mimetica*, specimen length: ~5mm (when stretched out as pictured here). B–*Elysia marcusi*, specimen length: ~4mm (when stretched out rather than disk-shaped as seen here). C–*Elysia tuca*, specimen length: ~7mm. D–*Elysia papillosa*, specimen length: ~8mm. E–*Elysia cornigera*, specimen length: ~7mm. F–*Elysia timida*, specimen length: ~8mm. G–*Ercolania fuscata*, specimen length: ~3mm. H–*Ercolania viridis* (both color morphs found in Florida), specimen length: ~3-4mm. I–*Placida dendritica*, specimen length: ~4mm. J–*Elysia patina*, specimen length: ~6mm. Photo by Gregor Christa, used with permission. K–*Thuridilla hopei*, specimen length: ~8mm.

*Elysia timida*, *Placida dendritica*, *Thuridilla hopei* and *Bosellia mimetica* adults were individually collected in May 2014/2015 from Fetovaia on the island of Elba, Italy. Stones covered in young *Acetabularia acetabulum* were also collected at the same locality. *Elysia marcusi*, *El*. *tuca*, *El*. *cornigera*, *El*. *papillosa*, *El*. *patina*, *Ercolania viridis* and *Er*. *fuscata* were all collected at Spanish Harbor Key and Long Key, Florida USA (Fig 1A–1K). All specimens were kept at either 18°C (Mediterranean specimens) or 21°C (Caribbean specimens) under full spectrum light (220 μmol quanta m^-2^ s^-1^) for 12h L: 12h D (day and night rhythm) and provided fresh water every 3 days. Table 1 further explains where each species was collected and the algae on which it was collected. Table 2 gives an overview of how many *El*. *timida* juveniles, from which age groups, were used for each of the experiments presented here. All adult animals were measured with a Diving PAM Fluorometer (Walz Heinz GmbH, Germany) at each time point during starvation and their starvation longevity was recorded. Juvenile animals were too small to be measured with this device.

**Table 1:** Specimen collection information.

Species	# Used in Starvation Experiments	Collection Location	Associated algae	Collection Notes
*Bosellia mimetica*	10	Mediterranean–Elba–Fetovaia Bay (April 2014)	*Halimeda tuna*	Many differently sized individuals found in high abundance on *H*. *tuna* from 1-15m depth.
*Elysia cornigera*	10	Caribbean–Florida Keys, USA–Spanish Harbor Key (April 2016)	*Acetabularia penniculus*	Found on subtidal rocks in *A*. *penniculus* meadows, 0-2m depth. Observed feeding on *A*. *penniculus*.
*Elysia marcusi*	10	Caribbean–Florida Keys, USA–Long Key, Bayside (April 2016)	*Halimeda opuntia*	Many differently sized individuals found on *H*. *opuntia* from 1-4m depth.
*Elysia papillosa*	8	Caribbean–Florida Keys, USA–Spanish Harbor Key (April 2016)	*Acetabularia penniculus*	Found on subtidal rocks in *A*. *penniculus* meadows, 0-2m depth. Not observed feeding on *A*. *penniculus*.
*Elysia patina*	10	Caribbean–Florida Keys, USA–Spanish Harbor Key (April 2016)	*Acetabularia penniculus*	Found on subtidal rocks in *A*. *penniculus* meadows, 0-2m depth. Not observed feeding on *A*. *penniculus*.
*Elysia timida*	see Table 2	Mediterranean–Elba–Fetovaia Bay (April 2014)	*Acetabularia acetabulum*	Found on subtidal rocks in *A*. *acetabulum* meadows 0-5m depth. Observed feeding on *A*. *acetabulum*.
*Elysia tuca*	10	Caribbean–Florida Keys, USA–Spanish Harbor and Long Keys (April 2016)	*Halimeda opuntia*, *H*. *macroloba*	Many differently sized individuals found in high abundance on *Halimeda* sp. from 1-10m depth.
*Ercolania fuscata*	10	Caribbean–Florida Keys, USA–Spanish Harbor Key (April 2016)	*Cladophora liniformis* cf?	Found on large *C*. *liniformis* mats covering rocks from 0–0.3m depth.
*Ercolania viridis*	8	Caribbean–Florida Keys, USA–Long Key Bayside (April 2016)	Either *Halimeda* sp. or *Avrainvillea* sp.	Found in tanks containing *Halimeda* sp. and *Avrainvillea* sp. Both algal species were collected at 2-3m depth.
*Placida dendritica*	10	Mediterranean–Elba–Fetovaia Bay (April 2014)	*Codium fragile*	Found on *C*. *fragile* at 2-18m depth. Observed feeding on *C*. *fragile*.
*Thuridilla hopei*	10	Mediterranean–Elba–Fetovaia Bay (April 2014)	unknown	0-15m depth. Found in *A*. *acetabulum* meadows and deeper rocks. Not observed feeding on *A*. *acetabulum*.

**Table 2:** Overview of each experiment detailing the number and age of the juvenile *El*. *timida* used in this study. Parentheses refer to animals that were included at the beginning of the experiment and placed in starvation, but did not survive until the date they were to be surveyed. They are listed here for clarity even though they did not survive long enough to be used in the experiments and were therefore excluded from all analyses. DPM–Days post-metamorphosis.

Experiment number and Number and description	Time points surveyed (after starvation begins)	Number of 4 DPM juveniles used	Number of 10 DPM juveniles used	Number of 15 DPM juveniles used	Number of 25 DPM juveniles used
1 Juvenile longevity without handling	Every specimen, every day until death	10	10	10	10
2 Chlorophyll *a* degradation	Every specimen, every day until death	10	10	10	10
3 Digestive activity at different time points in starvation	0 days starved (control)	3	3	3	3
	3 days starved	(3)	3	3	3
	7 days starved	(3)	3	3	3
	10 days starved	(3)	(3)	3	3
	15 days starved	(3)	(3)	(3)	3
	25 days starved	(3)	(3)	(3)	3
4 Food reintroduction experiment: Starved juveniles (S) and Starved then Reintroduced to Food (S+RF) juveniles	0 days starved (control)	6 total, 3 S and 3 S+RF	6 total, 3 S and 3 S+RF	6 total, 3 S and 3 S+RF	6 total, 3 S and 3 S+RF
	3 days starved	(6) total, (3) S and (3) S+RF	6 total, 3 S and 3 S+RF	6 total, 3 S and 3 S+RF	6 total, 3 S and 3 S+RF
	7 days starved	(6) total, (3) S and (3) S+RF	6 total, 3 S and 3 S+RF	6 total, 3 S and 3 S+RF	6 total, 3 S and 3 S+RF
	10 days starved	(6) total, (3) S and (3) S+RF	(6) total, (3) S and (3) S+RF	6 total, 3 S and 3 S+RF	6 total, 3 S and 3 S+RF
	15 days starved	(6) total, (3) S and (3) S+RF	(6) total, (3) S and (3) S+RF	(6) total, (3) S and (3) S+RF	6 total, 3 S and 3 S+RF
	25 days starved	(6) total, (3) S and (3) S+RF	(6) total, (3) S and (3) S+RF	(6) total, (3) S and (3) S+RF	6 total, 3 S and 3 S+RF

To obtain juveniles for the experiments, adult *El*. *timida* were allowed to feed freely, their egg masses were removed from the stones or tank walls and cultivated separately in small dishes. Embryos hatched after 21–24 days in the egg mass as shelled larvae (Type 2 according to Thompson and Brown 1976). They metamorphosed, discarding their shells after 3–5 days. Directly after metamorphosing, they were presented with food and were allowed to feed for differing lengths of time before experimentation began: 4, 10, 15 and 25 Days Post-Metamorphosis (DPM). These intervals were chosen to surround the 12-day time point reported by Marín and Ros [1993].

Juvenile slugs of each age (4, 10, 15, 25 DPM) were used in four different experiments. The number of specimens and overview of each experiment is detailed in Table 2. For each experiment, animals were placed in a separate bowls without access to algae. In the first experiment (juvenile survival without handling, see Table 2), ten animals were selected from each age group (4, 10, 15, 25 DPM) and monitored during starvation as a control to the various handling, e.g., with MgCl_2_ and /or cooling, effects on juvenile viability. They were examined under a microscope daily, in their individual bowls while starving, to assess vitality. This revealed the base rate of starvation for each age group. Their longevity was recorded and compared to those in the Experiments 2 to 4, as outlined in Table 2.

In the second experiment, another ten animals for each time point, were imaged daily under the confocal microscope in order to observe chlorophyll *a* degradation (see Table 2). They were first removed from the algae and placed in 7% MgCl_2_ for up to three hours until they stopped moving. The animals that did not stop were then cooled until movement slowed enough for imaging. Once still, they were transferred to slides containing a well. They were then scanned using the blue laser 488nm excitation (600-640nm accepted emission range, chl *a* optimum: 633) on a Leica SPE confocal laser scanning microscope. The entire animal was scanned in order to view any chlorophyll *a* autofluorescence present. They were then returned to individual bowls. This process was repeated every day for each animal until death.

In a third experiment, lysosomal abundance and activity (during starvation) were recorded by staining 18 animals from each age group (allowed to feed for 4, 10, 15, 25 Days Post-Metamorphosis DPM), with Acridine Orange, a fluorescent stain that aggregates in extremely acidic regions (pH < 4.5) [46,49,50] (Table 2). As Acridine Orange stains acidic organelles in living tissues, living animals were first stained for 30 minutes at room temperature (diluted with filtered seawater to a 5μmol solution) and then vivisected, mounted and imaged on the same microscope using the blue laser (excitation 488nm), with 645-670nm (AO dimer type optimum: 656) as the accepted emission range [46]. These animals were also examined for their functional chloroplast abundance. Three animals were investigated at the following time points: 1, 3, 7, 10, 15 and 25 days in starvation, although the number of days survived for the younger juveniles was very low and increased in number with increasing age of juveniles (see Table 2).

The final (fourth) experiment comprised individuals from each age group that were first starved and then re-introduced to food before Acridine Orange staining to determine the increase in chlorophyll *a* gained by one feeding and explore any changes in digestive activity when reintroduced to food. They were not subjected to MgCl_2_, but were cooled before imaging. Six specimens were placed in starvation for each point surveyed. Three individuals were measured at each starvation time point (Table 2, S), while the other the three specimens were starved under the same condition but received food for 2 hours directly before being measured (Table 2, S+R). Staining began directly after feeding and lasted 30 minutes, so the observed lysosome abundances provided enough time for both chloroplast uptake and cellular response to newly sequestered kleptoplasts [19].

Image stacks were analyzed using the Fiji/ImageJ plugin, 3D-AMP [46]. Lysosome and chloroplast abundance were estimated using the area measurements provided by the FIJI–ImageJ Plugin, 3D-AMP [46]. Normality for each experiment was assessed using a Kolmogorov-Smirnov test, since some of the samples contained many identical values. After confirming normality, two-tailed T-tests were used to compare the means in each treatment, for each age group.

## Results

### Handled juveniles compared to un-handled specimen longevity (experiment #1 & 2)

The juveniles imaged in these experiments were always alive when imaged, so steps were taken to reduce sample movement and therefore the error associated with it. This involved first subjecting each animal to a 7% MgCl_2_ and seawater mixture and then cooling each slide on a block of ice until movement stopped (usually 2–3 minutes). MgCl_2_ did not appear to affect the mobility of juveniles younger than 25-days-old when they began the starvation period, although it did slow many of the animals after 7 days of starvation. The 25 DPM animals were always slowed, if not completely immobilized by the MgCl_2_. Higher concentrations were not applied due to any potential toxic effects, and juveniles treated with lower concentrations showed no decrease in movement.

To determine any detriment to the slugs’ longevities, a separate population, containing each of the age groups was not treated with MgCl_2_, put on ice nor imaged. The longevities of these animals were recorded, and while they appear to live slightly longer than the treated animals in each group, two-tailed T-Tests reveal no significant difference in the life span of the animals in each group (4 DPM slugs: p = 1, 10 DPM slugs p = 0.88, 15 DPM slugs p = 0.71 and 25 DPM slugs p = 0.53. Each treatment was normally distributed according to a Kolmogorov-Smirnov test: 4 DPM max difference [md]– 0.08 (critical difference [cd]– 0.27); 10 DPM md– 0.23 (cd– 0.25); 15 DPM md– 0.14 (cd– 0.18); 25 DPM md– 0.03 (cd– 0.09).

### Juvenile *Elysia timida* longevity (experiment #2)

After hatching, *El*. *timida* veliger larvae were presented with *Acetabularia acetabulum*, although they were not observed feeding for 1–3 days until they crawled out of their larval shells. Once in starvation, many of these animals died with intact chloroplasts filling their digestive glands. The 4 DPM juveniles starved for an average 1.4 ± 0.84 days, with one animal dying within the first 24 hours and the longest surviving animal reaching 3 days. The 10 DPM population survived an average 2.3 ± 1.57 days, having 2 animals dying within the first 24 hours and 5 days as a maximum starvation time. Of the 15 DPM animals, the shortest lifespan was 3 days, the average 5.6 ± 2.01 days and the maximum 9 days. The 25 DPM juveniles reached a maximum starvation time of 22 days, and average of 18.4 ± 2.17 days and a minimum of 14 days.

**Fig 2:**
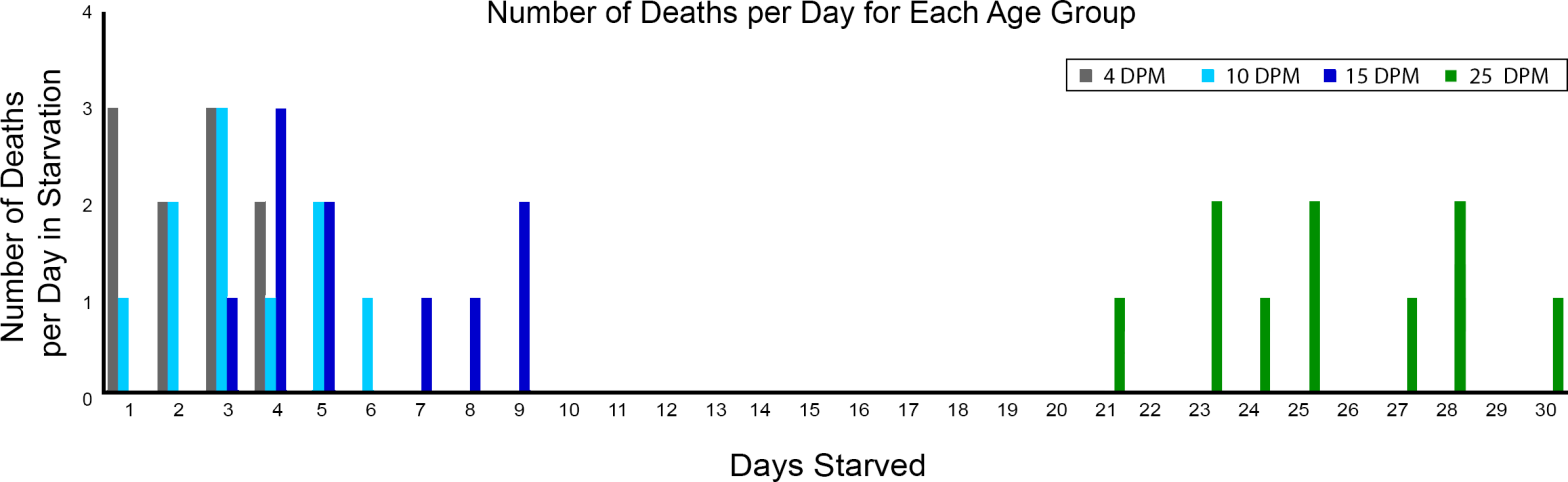
The number of deaths per day in starvation. Individuals from each age group (4, 10, 15, 25 DPM) were monitored each day until death. 4 DPM deaths are indicated in grey, 10 DPM deaths in light blue, 15 DPM in dark blue and 25 DPM in green. The relatively even distribution suggests a range of fitness levels amongst specimens of each age. The large gap depicts the differences between the younger time points (4, 10, 15) where functional kleptoplasty is not occurring and the 25 DPM juveniles where short-term retention has been developed.

The number of deaths per day was also recorded, with no more than 3 deaths occurring on a single day in any of the age groups, and a multi-day span when each group’s deaths occurred. The 4 DPM group died throughout a four-day time span, the 10 DPM group spanned six days, from one death in the first 24 hours to the sixth starvation day (Fig 2). The 15 DPM deaths were spread over seven days (the first occurring after three days and the last on the ninth day) and the 25 DPM animals spanned ten days (starvation days 21–30) (Fig 2). Comparing the average life span of each age group with a Tukey HSD test revealed significant differences between 4 and 15 DPM juveniles (p = 0.006), 4 and 25 DPM juveniles (p = 0.001), 10 and 25 DPM juveniles (p = 0.001), 15 and 25 DPM juveniles (p = 0.001). No significant difference was revealed between 4 and 10 DPM individuals (p = 0.71), and 10 and 15 DPM juveniles (p = 0.08).

### Chloroplast abundance and degradation in juvenile *El*. *timida* (experiment #2)

**Fig 3:**
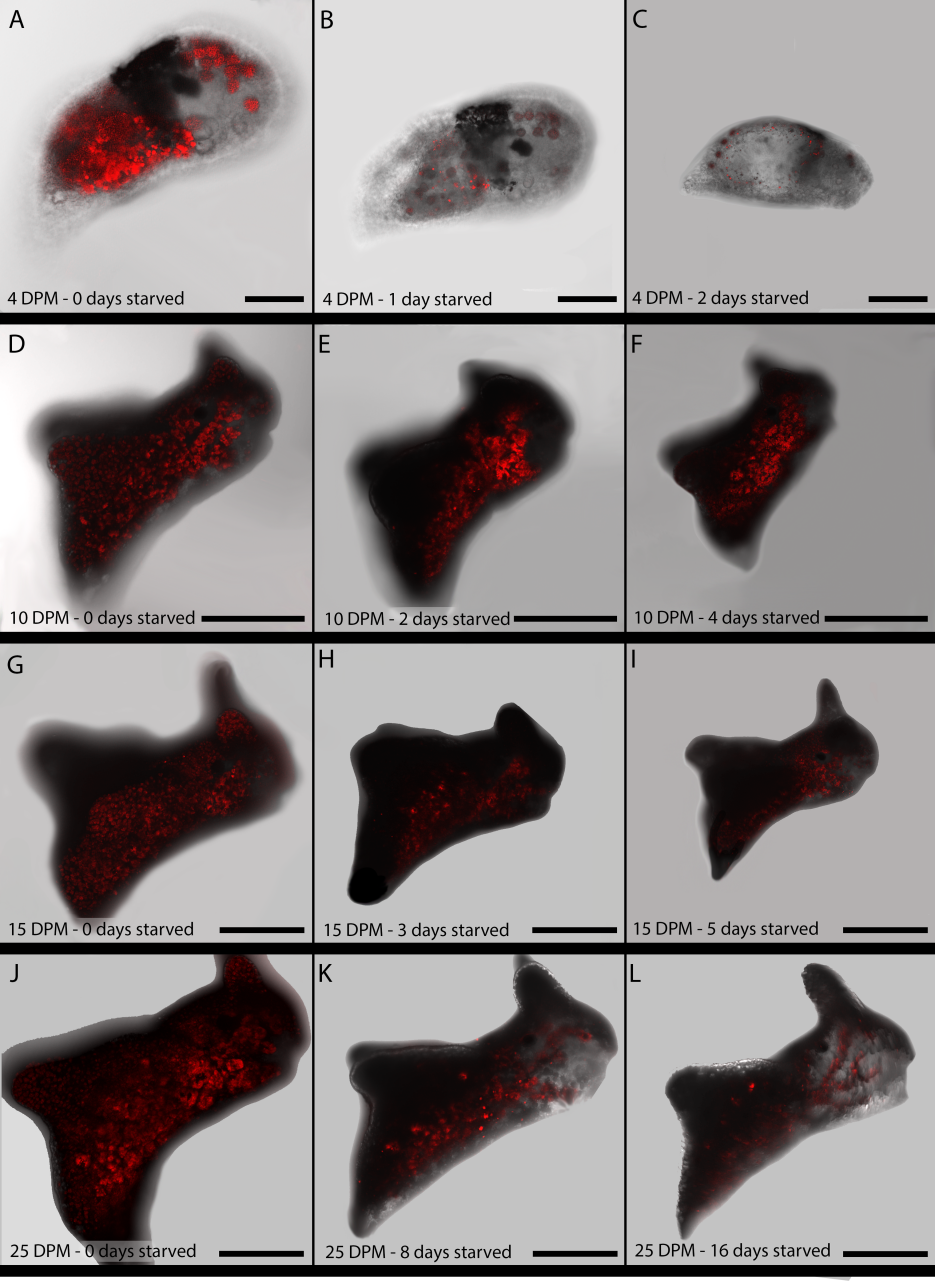
Confocal microscopy images of juvenile *El*. *timida* and their chlorophyll *a* content during starvation. A– 4 DPM juvenile before starvation (day 0). B–The same 4 DPM juvenile after 1 day of starvation. C–The same 4 DPM juvenile after 2 days of starvation. D– 10 DPM juvenile before starvation (day 0). E–The same 10 DPM juvenile after 2 days of starvation. F–The same 10 DPM juvenile after 4 days of starvation. G– 15 DPM juvenile before starvation (day 0). H–The same 15 DPM juvenile after 3 days of starvation. I–The same 15 DPM juvenile after 5 days of starvation. J– 25 DPM juvenile before starvation (day 0). K–The same 25 DPM juvenile after 8 days of starvation. L–The same 25 DPM juvenile after 16 days of starvation. Scale bars: A-C 70μm; D-F 250μm; G-I 350μm, J-L: 450μm.

**Fig 4:**
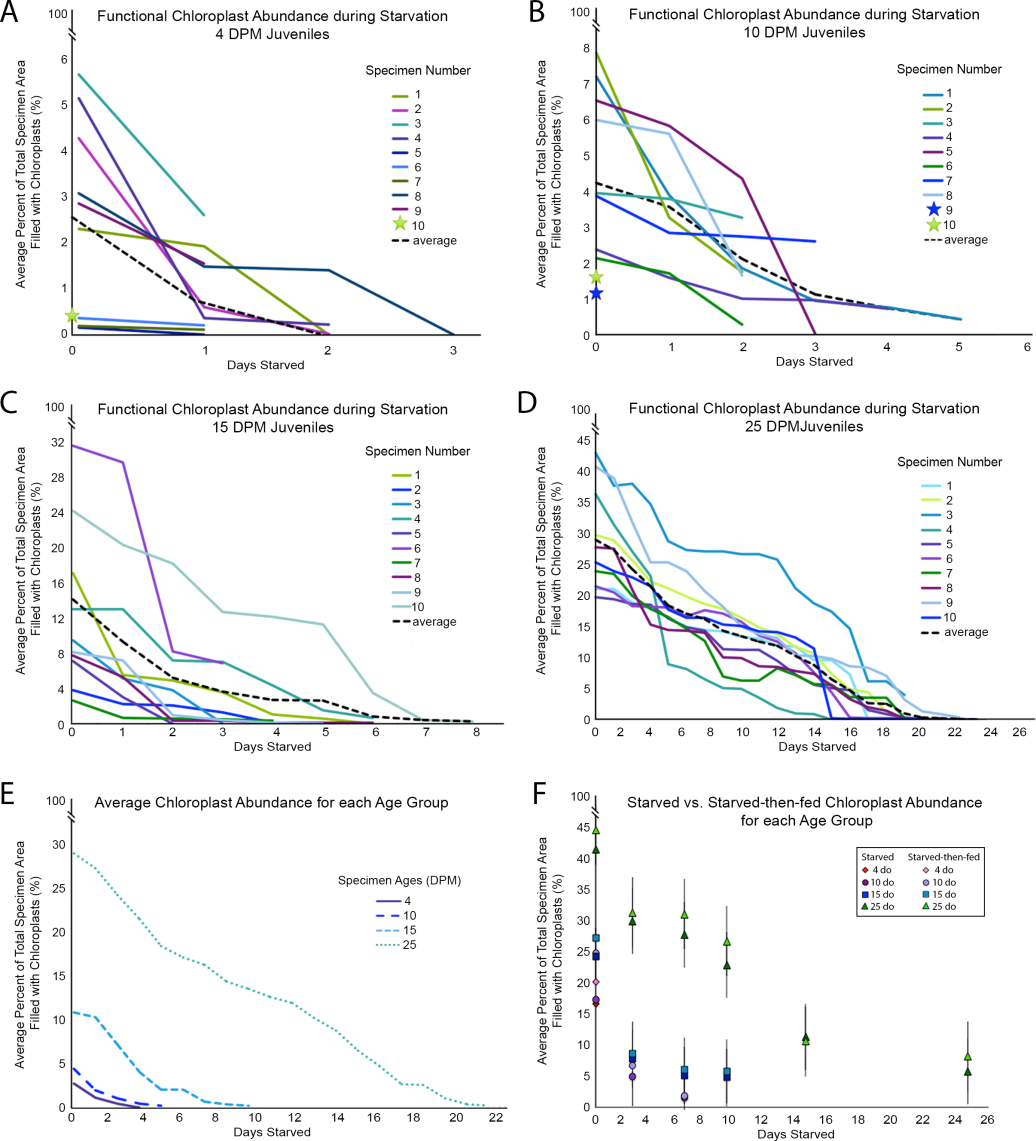
Functional chloroplast abundance during starvation for each age group (DPM). The average juvenile longevity is shown by the black dotted lines. A–Each juvenile *El*. *timida* (n = 10), aged 4 days post-metamorphosis (DPM) was measured daily to determine the decrease in functional chloroplast abundance throughout the starvation period until death. The green star indicates an animal that died within the first 24 hours and could not be represented by a line. B–The longevity of each juvenile *El*. *timida* specimen aged 10 DPM (n = 10). The green and blue stars indicate individual animals that died within the first 24 hours and could not be represented by a line. C– 15 DPM juvenile longevities in starvation (n = 10). D– 25 DPM individual longevities in starvation (n = 10). E–The average chloroplast abundance for each age group depicted in A-D, plotted together for comparison with the same axes scaling. F–Functional chloroplast abundance in starved and starved then reintroduced to food *El*. *timida* juveniles of each age group for each time point surveyed. Juveniles of each age group were starved and their functional chloroplast abundances recorded at 0, 3, 7, 10, 15 and 25 in starvation. Additional animals were re-introduced to food and allowed to feed for 2 hours before their chloroplast abundances were recorded. 4 DPM juveniles are indicated by diamonds: red for starved and pink for starved-then-fed; 10 DPM juveniles are shown by circles: purple for starved and lavender for starved-then-fed; 15 DPM juveniles are denoted by squares, dark blue for starved and light blue for starved-then-fed; 25 DPM juveniles are designated with triangles: dark green for starved and light green for starved-then-fed.

Ten juveniles from each age group were imaged daily with the CLSM, to determine the decline in functional chloroplasts within each animal every day (Fig 3A–3L). Chloroplast abundance was assessed using 3D-AMP, a FIJI plugin that reports the number of pixels in an image stack, so the final chloroplast abundances are reported here in percent coverage areas: the area covered by functional chloroplasts compared to the animal’s overall area. The 4 DPM juveniles showed varying percent coverage, with 4 animals having a mean 0.3% and the rest ranging from 2.2–5.8% on the day they began starving (Fig 4A). Each 10 DPM animal ranged from 1.2–8% (Fig 4B), the 15 DPM animals ranged from 3–30.8% (Fig 4C) and the 25 DPM animals spanned 19–43% chloroplast coverage at the beginning of the starvation period (Fig 4D). While some animals had 0% chloroplast coverage upon death, many died with undigested functional kleptoplasts within their bodies. Six 4 DPM slugs and all 10 DPM slugs fall into this category, whereas the older age groups (15- and 25- day-old) each only had 1 animal die containing functional kleptoplasts–every other animal was devoid of functional chloroplasts. The average kleptoplast coverage throughout the starvation period for each group is shown in Fig 4E.

### Juvenile digestion and lysosomal activity (experiment #3)

**Fig 5:**
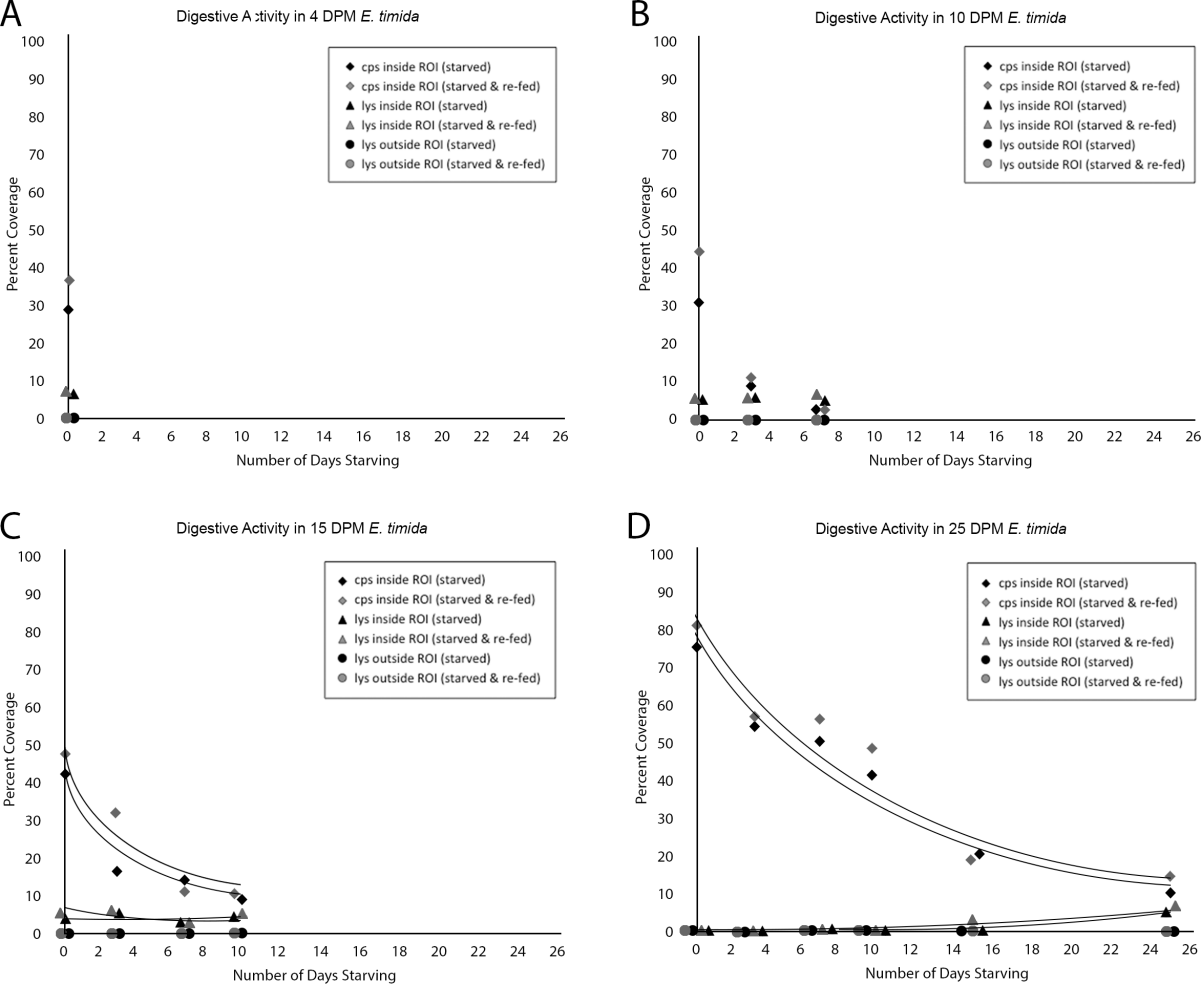
Digestive activity as indicated by chloroplast and lysosome abundance in specimens of 4, 10, 15 and 25 Days Post-Metamorphosis (DPM) *El*. *timida*. Black shapes indicate starved animals and grey shapes indicate starved-then-fed animals. Diamonds show chloroplast abundance inside the digestive gland tubule, here referred to as the region of interest (ROI), triangles denote lysosome abundance inside the ROI and circles depict lysosome abundance outside the ROI. All values are given in percent coverage, the percentage of tissue either inside or outside the ROI where fluorescent signal was measured as compared to the total area covered by the tissue. Overlapping shapes were illustrated directly next to each other for clarity, but were measured on the same day. A–digestive activity in 4 DPM juveniles. B– 10 DPM juveniles. C– 15 DPM juveniles. D– 25 DPM juveniles.

Lysosomal abundances inside and outside the digestive gland tubules were measured for each of the age groups at the following time points: 0, 3, 7, 10, 15, and 25 days when they survived long enough to be measured. Inside the digestive gland tubule, lysosomal activity serves as an indicator of intracellular digestion, whereas outside the digestive gland tubule, lysosomal activity may indicate autophagy. The 4 DPM slugs were only measured at one time point (0-days), since they did not starve long enough to be measured at the three-day time point. Since both the 4- and 10 DPM juveniles had less than three measurements, no best-fit curves could be applied. The average lysosome coverage for 4 DPM slugs was 6.38 ± 0.8% in the digestive gland tubule (DGT), and 0.11 ± 0.1% outside the DGT (Fig 5A). The average coverage within the DGT for 10-day old animals was relatively constant throughout the starvation period, at 5.5 ± 1%, and was always between 0 and 1% outside the DGT (Fig 5B). Starving 15 DPM slugs had slightly more fluctuating lysosomal coverage, starting at 4.21 ± 2.1%, dipping at 7 days of starving to 2.6 ± 0.9% and then returning to 4.5 ± 0.2%. Outside the DGT, their percent coverage was close to 0 (Fig 5C). Only the 25 DPM animals started the starvation period with very low values inside the DGT (0.2%), which stayed low until 15 days in starvation when it rose to 2.3 ± 0.7% and finally 5.6 ± 1.3% at the 25-day time point (Fig 5D).

### Re-fed *El*. *timida* juveniles (experiment #4)

Six juvenile slugs from each age group were starved (Table 2 (S)) and three of them were then reintroduced to food (Table 2 (S+R)) before their chloroplast and lysosome abundances were imaged to determine the increase in chloroplasts after a single feeding. Lysosomal activity of starved and re-fed specimens (S+F) were compared to those that were only starved (S). A Kolmogorov-Smirnov test revealed normally distributed data for each group compared. In the 4 DPM unstarved slugs (control), there were significantly more chloroplasts inside the digestive gland tubules (DGT), (S: 29.9%, S+R: 36.2%) (two-tailed T-test: p = 0.005), however lysosome abundance inside and outside the DGT were not significantly different in the slugs provided with food after starving when compared to the un-fed slugs (S: 6.4%, S+R: 6.9%; two-tailed T-test: p = 0.4 lysosomes in DGT), (S: 0.2%, S+R: 0.2%; two-tailed T-test: p = 0.66 lysosome outside DGT) (Fig 5A).

The 10 DPM unstarved slugs followed a similar trend, having no significant difference in the lysosome abundance outside or inside the DGT (S: 0.7%, S+R:0.8%; p = 0.88 and S: 5.9%, S+R:6.1%; p = 0.75 respectively) and a significant difference in chloroplast abundance (S: 31.1%, S+R:44.6%; p = 0.01). After 3 days of starvation however, the chloroplast difference after feeding decreases and is no longer significant (S: 9.7%, S+R: 10.1%; p = 0.1) and the lysosome values remain insignificant (S: 6.0%, S+R: 6.1% inside DGT; p = 0.59 and S: 0.8%, S+R: 0.9%; p = 0.43 outside DGT) (Fig 5B).

The first two measurements (unstarved and 3 days starving) for 15 DPM slugs had significant differences in the number of functional chloroplasts when comparing slugs that were allowed to feed to those not given food (S: 43.5%, S+R: 49.0%; p = 0.05 unstarved and S: 17.2%, S+R: 32.5%; p = 0.001 after 3 days). Lysosomal abundances inside and outside the digestive gland tubule were never significant (S: 8.2%, S+R: 8.4%; p = 0.74 inside DGT and S: 0.3%, S+R: 0.2%; p = 0.81 outside DGT) (Fig 5C). The same trend was seen in 25 DPM slugs, although the first 4 measurements had significant differences in the number of chloroplasts (unstarved S: 74.2%, S+R: 81.8%; p = 0.03; 3 days S: 54.9%, S+R: 57.8%; p = 0.05; 7 days S: 50.5%, S+R: 57.2%; p = 0.001, 10 days S: 41.7%, S+R: 48.8%; p = 0.01). At 15 and 25 days of starvation though, the number of chloroplasts were no longer significant (S: 20.8%, S+R: 19.7%; p = 0.78 and S: 10.3%, S+R: 13.6%; 0.59 respectively). The difference in lysosome abundance inside and outside the DGT also lacked significance (Fig 5D). The trends for each group are summarized in Fig 4F.

### Comparison to non-retaining and short-term retaining slug species

Five short-term kleptoplast retaining species (StR), and five non-retaining (NR) species were measured with the Pulse Amplitude Modulated Fluorometer (PAM) to determine their photosynthetic activity during starvation and their longevity in starvation. The StR and NR designation were assigned according to published reports [5,48]. Since this study uses these NR and StR species to provide context for the argument that functional kleptoplasty develops after 15 days in juvenile *El*. *timida*, we wanted to verify that the specimens observed here had photosynthetic activities similar to those recorded in previous reports and be able to state any discrepancies.

**Fig 6:**
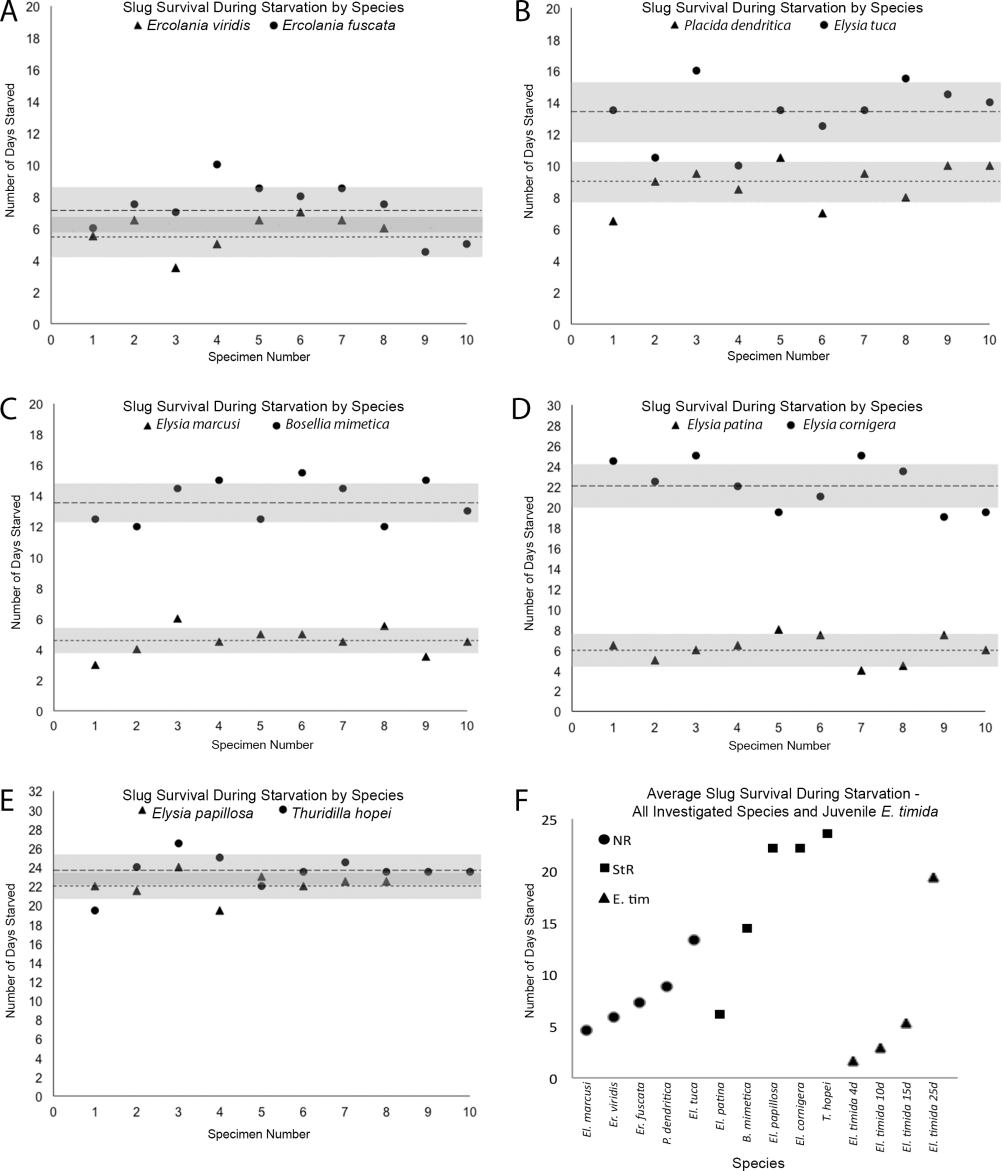
Longevity in starvation by species. A-E–The x-axis shows the individual specimen number and the y-axis records the number of days that specimen survived. The black dotted and dashed lines show the respective averages for each species and the grey bars indicate one standard deviation to each side of the mean. A–*Ercolania viridis* is denoted by triangles and *Ercolania fuscata* by circles. The *Er*. *viridis* average was 5.8 ± 1.1 days (dotted line) and *Er*. *fuscata* averaged 7.25 ± 1.7 (dashed line). B–*Placida dendritica* is represented by triangles and *Elysia tuca* by circles. The *P*. *dendritica* average was 8.85 ± 1.3 days (dotted line) and *El*. *tuca* averaged 13.35 ± 1.9 (dashed line). C–*Elysia marcusi* is signified by triangles and *Bosellia mimetica* by circles. The *El*. *marcusi* average was 4.55 ± 0.89 days (dotted line) and *B*. *mimetica* averaged 13.65 ± 1.4 (dashed line). D–*Elysia patina* is conveyed by triangles and *Elysia cornigera* by circles. The *El*. *patina* average was 6.15 ± 1.7 days (dotted line) and *El*. *cornigera* averaged 22.15 ± 2.3 (dashed line). E–*Elysia papillosa* is designated by triangles and *Thuridilla hopei* by circles. The *El*. *papillosa* average was 22.1 ± 1.3 days (dotted line) and *T*. *hopei* averaged 23.55 ± 1.8 (dashed line). F–All of the short- and non-retaining species average longevities in starvation and the *El*. *timida* juveniles average longevities are depicted, showing the clear difference in juvenile longevities and grouping within either the short-term retention slugs and non-retaining slugs. Circles depict Non-Retaining slug species (NR); squares indicate Short-term Retaining (StR) species and triangles show the different *El*. *timida* juvenile age groups.

**Fig 7:**
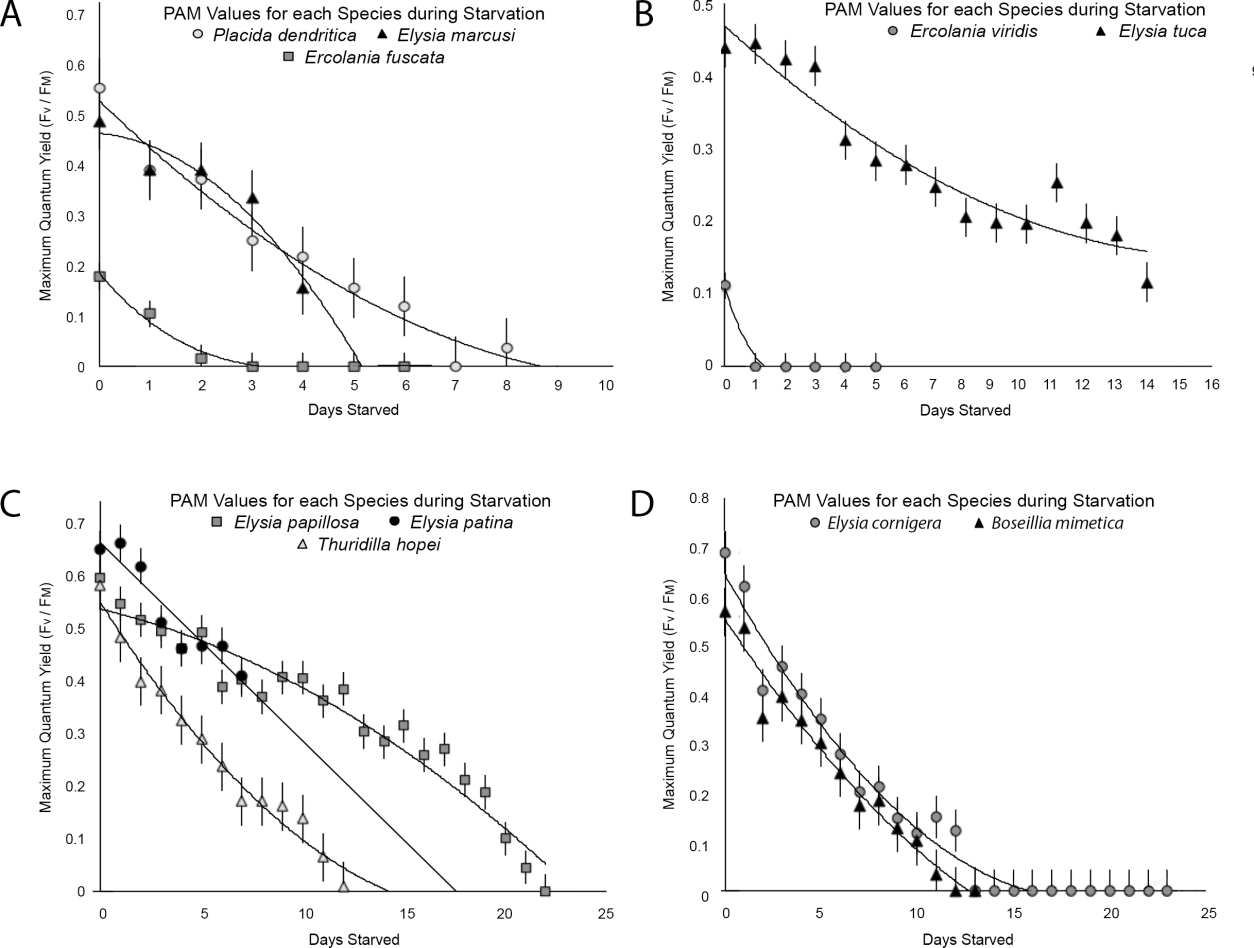
Pulse Amplitude Modulated (PAM) fluorometry values for a range of short-term retention and non-retaining species during starvation. A–The PAM values for *Placida dendritica* are indicated by light grey circles, *Elysia marcusi* by black triangles and *Ercolania fuscata* by dark grey squares. *P*. *dendritica* is best modeled by the function y = 4.22x^2^–103.95x + 610.26 (R^2^ = 0.97), *El*. *marcusi* by y = -15.17x^2^ + 21.83x + 441 (R^2^ = 0.92) and *El*. *fuscata* by y = -1.59x^3^ + 28.87x^2^–169.65x + 320.76 (R^2^ = 0.98). B–The PAM values for *Ercolania viridis* are indicated by grey circles and *Elysia tuca* by black triangles. *Er*. *viridis* is best modeled by the function y = -5.14x^3^ + 63.87x^2^–248.13x + 296 (R^2^ = 0.95) and *El*. *tuca* by y = 1.14x^2^–40.43x + 507.49 (R^2^ = 0.91). C–The PAM values for *Elysia papillosa* are indicated by dark grey squares, *Elysia patina* by black circles and *Thuridilla hopei* by light grey triangles. *El*. *papillosa* is best modeled by the function y = -0.55x^2^–7.54x + 514.6 (R^2^ = 0.94), *El*. *patina* by y = -35.59x + 660.95 (R^2^ = 0.89) and *T*. *hopei* by y = 1.48x^2^–60.38x + 577.04 (R^2^ = 0.98). D–The PAM values for *Elysia cornigera* are indicated by grey circles and *Bosellia mimetica* by black triangles. *El*. *cornigera* is best modeled by the function y = 1.91x^2^–74.94x + 718.98 (R^2^ = 0.97) and *B*. *mimetica* by y = 1.26x^2^–62.16x + 615.03 (R^2^ = 0.97)The error bars show the standard error.

NR species *Ercolania viridis* (n = 8) and *Ercolania fuscata* (n = 10) starved an average of 5.8 ± 1.1 days and 7.25 ± 1.69 days respectively (Fig 6A). Both species had measurable average photosynthetic activity values (F_v_/F_M_) on the first day they were measured, *Er*. *viridis* with 0.111 (y = -5.14x^3^ + 63.87x^2^–248.13x + 296, R^2^ = 0.95) and *Er*. *fuscata* had 0.173 (y = -1.59x^3^ + 28.87x^2^–169.65x + 320.76, R^2^ = 0.98), however after 24 hours, no autofluorescence was measured in either species (Fig 7A and 7B). *Elysia marcusi* (n = 10) averaged 4.5 ± 0.9 days in starvation (Fig 6C), while *Elysia tuca* (n = 10) and *Placida dendritica* (n = 10) starved longer, with 13.3 ± 1.9 and 8.6 ± 1.3 days respectively (Fig 6B). Photosynthetic activity was measured in unstarved specimens for each species: *El*. *marcusi* having an F_v_/F_M_ = 0.471 on the first day in starvation and 0 by the fifth day (y = -15.17x^2^ + 21.83x + 441, R^2^ = 0.92) (Fig 7A); *El*. *tuca* starting starvation with F_v_/F_M_ = 0.438 and continuing to have photosynthetic activity after death (y = 1.1433x^2^–40.434x + 507.49, R^2^ = 0.91) (Fig 7B), and *P*. *dendritica* which began starvation with an F_v_/F_M_ = 0.533 and showed little to no photosynthetic activity upon death (0.0–0.1) (y = 4.22x^2^–103.95x + 610.26, R^2^ = 0.97) (Fig 7A).

StR species *Bosellia mimetica* survived 13.7 ± 1.4 days (n = 10) in starvation, starting starvation with an F_v_/F_M_ = 0.567 and ending with 0.0 (y = 1.26x^2^–62.16x + 615.03, R^2^ = 0.97) (Figs 6C and 7D). *Elysia cornigera* had an F_v_/F_M_ = 0.688 when unstarved and 0.0 after 14 days, despite living an average 22.1 ± 2.3 days (n = 10) (y = 1.9132x^2^–74.939x + 718.98, R^2^ = 0.97) (Figs 6D and 7D). *Elysia patina* started starvation with F_v_/F_M_ = 0.614, ending after an average 6.1 ± 1.7 days (n = 10) with F_v_/F_M_ = 0.386 (y = -35.591x + 660.95, R^2^ = 0.88) (Figs 6D and 7C). *Elysia papillosa* starved an average of 22.1 ± 1.3 (n = 8), having a beginning F_v_/F_M_ = 0.562 and ending at 0.0 after 22 days (y = -0.553x^2^–7.5443x + 514.6, R^2^ = 0.94) (Figs 6E and 7C). *Thuridilla hopei* (n = 10) began starvation with F_v_/F_M_ = 0.548, with no detectable autofluorescence after 13 days, despite surviving an average 23.6 ± 1.8 days (y = 1.4795x^2^–60.376x + 577.04, R^2^ = 0.97) (Figs 6E and 7C). The average longevity in starvation for each species and the different *El*. *timida* age groups is summarized in Fig 6F.

## Discussion

*Elysia timida* is increasingly used as a model organism in the search to understand the development and evolution of functional kleptoplasty since it is commonly found and only feeds on one algal species, which limits experimental uncertainty due to multiple incorporated kleptoplast species [9,11,15,16,35,46]. *El*. *timida* juveniles are incapable of retaining functional chloroplasts, showing that this ability must be developed as the young slug develops. Although Marín and Ros (1993) state that functional kleptoplasty is established after 12 days, they do not provide evidence supporting this claim. The longevity experiments conducted here dispute this claim, instead showing functional kleptoplasty is likely developed more than 15 days after metamorphosis and shell loss–maybe even after 25 days, see below, when algae is continuously available for feeding. This discrepancy may be due to population differences, temperature or a host of other abiotic and biotic factors.

### Juvenile handling with and without MgCl_2_ and cold (Experiments 1 & 2)

The exposure to cold and MgCl_2_ did not affect juvenile longevity and in most cases, did not seem to affect the animal’s locomotion. Both of these techniques are frequently used to immobilize living animals but they both had little effect on juvenile *El*. timida despite functioning in adults [51–53]. They also did not affect the longevity of the animal, as the average longevities for the treated animals showed no significant difference to animals that were untreated.

### The development of functional kleptoplasty in juvenile *El*. *timida*

Only one other sacoglossan species has been investigated regarding larval development and the acquisition of functional kleptoplasty, the LtR, heterokontophyte feeding species *Elysia chlorotica*. Pelletreau et al. [36,42] reported post-metamorphic juveniles requiring 7 days of feeding before they gained the ability to retain functional kleptoplasts and defined the term transient kleptoplasty in reference to the time period when kleptoplasty is being established but does not function as observed in adults. This conclusion is based on laboratory cultured *El*. *chlorotica* that were photographed until 10 DPM. Our study shows that *El*. *timida* needs more than 15 days, suggesting each LtR species gains this ability at a different time and may even gain it through a different process although this will take further investigation to confirm. As previously shown for *E*. *chlorotica* [42], the data presented here indicates a transient kleptoplasty phase in *E*., *timida*, after the 15 DPM time point.

This argument is based on two of the experiments presented here. The longevity experiments show juveniles that are 4, 10 or 15 DPM as having very short longevities (a maximum 8 days) in starvation. While these animals survive shorter time periods than seen in adult non-retention forms, the starvation time for 25 DPM *El*. *timida* juveniles is well above all of the non-retaining slugs (NR), and is well within the range of the short-term retention (StR) slugs observed in this investigation, at a maximum 22 days. This is still only around one fourth of the total starvation survival duration accomplished by adult *E*. *timida*, (89 days) [9,46]. It is also important to note that the NR and StR forms surveyed here were adult slugs, and had significantly more body mass than the *El*. *timida* juveniles in our experiments here.

Some previous reports on longevity in starvation are similar to the results presented here (*El*. *patina*, *El*. *papillosa* [48]), while others present discrepancies suggesting a variability in starvation longevity that may be related to factors such as time of the year, light intensity, temperature, size of the investigated specimens or the number of individuals surveyed. Christa et al. [48] reported *El*. *tuca* capable of surviving 20 days in starvation although they only achieved 13.3 days here. *El*. *marcusi* survived 9 days in that study (referred to as “*Bosellia marcusi”*) but 4.5 days on average here and *El*. *cornigera* which was reported starving 12 days in their study and 22.1 days in this investigation. *P*. *dendritica* also starved more than 11 days in a previous report [47] but only 8.6 days here. Starvation longevity has not been previously assessed for the species, *Er*. *fuscata*, *Er*. *viridis*, *T*. *hopei* and *B*. *mimetica*, so this study constitutes the first report. Although this study appears to show a correlation between the duration functional chloroplasts remain in the slug’s digestive gland and the time it can withstand starvation (short-term retaining slugs outlive non-retaining species), the discrepancies described above and the lack of additional reporting on some species suggest that this conclusion should not be made without further and clarifying evidence. This sentiment aligns with previous work that has discussed this topic in detail [11,54].

### Functional kleptoplast abundance in juvenile *El*. *timida*

Although 25 DPM juveniles can withstand starvation longer than all of the NR slug species, many still died with functional chloroplasts in their digestive systems. Adult *E*. *timida* have been reported as either having or lacking functional kleptoplasts in their bodies after dying of starvation [16,35]. The number of kleptoplasts was drastically reduced compared to the number each individual starts starvation with, however the presence of functional kleptoplasts upon death indicates that not all kleptoplasts are digested and used to meet their energetic needs, even when they are about to die. This suggests the following options, that juvenile slugs are unable to digest all chloroplasts, that the energy required to digest these kleptoplasts is more than the juvenile can spare or that they take up more chloroplasts than they can use. Despite this, the decline in functional kleptoplasts indicates that *El*. *timida* juveniles of all ages (including the 25DPM specimens) are capable of digesting kleptoplasts. This agrees with transmission electron micrograph studies, where broken down kleptoplasts are seen within juvenile *El*. *timida* digestive tubules [9]. Other investigations have reported functional kleptoplasts in slugs that have died of starvation [16].

Two distinct groupings are seen within the 4 DPM juveniles. Four of these juveniles had very low amounts of chloroplasts in their bodies, indicating that they had fed less than the other juveniles in their age group. This difference may have affected their longevity, as they all died within the first 48 hours, however some of the juveniles with far higher chloroplast levels also died within this time span, so a direct comparison between longevity and chloroplast abundance cannot be drawn. The same can be said for each of the other age groups, since in each of the populations, there is no obvious advantage to having more chloroplasts in terms of days survived. This may indicate that these chloroplasts are not directly or fully contributing to meeting the slug’s nutritional demands throughout the starvation period, however possible contributions made by the accumulated photosynthates should not be ignored and require further investigation [35,43]. One 25 DPM specimen (Specimen #7, Fig 4D) showed a slight increase in chloroplast abundance at 11 days in starvation and this data point lacks a clear explanation since there are no reports of chloroplast division once incorporated. This is likely a random error and may be due to sample movement during scanning.

The overall percent coverage of chloroplasts in juvenile *El*. *timida* also suggests that transient kleptoplasty is established after 15 days of feeding, but before 25 days. The low percent coverage seen in 4, 10 and 15 DPM juveniles differs significantly from the high abundance seen in adults [46]. Only the 25 DPM juveniles reach the 35–70% coverage seen in the adults although not every 25 DPM juvenile fell within that range (19–43%). The overlap in some juveniles and some adult *El*. *timida’s* ranges here, (35–43%) indicates that at least some of these juveniles are filled with kleptoplasts to the degree with which adults are. This again aligns 25 DPM juveniles more with adults than with the other juvenile populations, despite their still immature morphology (the female gonad had not developed in any of the 25 DPM juveniles examined). This aligns with Schmitt et al. (2014) who stated that it takes 106 days for *El*. *timida* to develop a fully mature internal morphology and reproductive capabilities.

The variability of juvenile longevity in starvation suggests a range of individual fitness levels, consistent with an *r-*selection-species pattern, where many offspring are produced and very few actually survive. As *El*. *timida* egg clutches contain many offspring (19–249 according to Schmitt (2014)) and we observed many slugs hatching but not developing through metamorphosis, this finding is consistent with the predicted fitness strategy. The only fitness tests examined in these experiments screened out animals that did not hatch and those that couldn’t complete metamorphosis. Therefore, the animals used here were already amongst the fitter specimens, but following a successful metamorphosis, they were not additionally screened for size, weight, ingested kleptoplast content or other factors, meaning a variety of fitness levels should still be present. Despite this, no significant advantage was observed in any of the experiments, where one specimen drastically outlived the others in its age group. The low standard deviations surrounding the average longevities also suggest that while individual variability and fitness should be considered, it was not overly apparent that some individuals were better suited to survival in starvation.

### Digestive activity in *E*. *timida* juveniles

Lysosomal activity in 4, 10 and 15 DPM animals was relatively stable, with around 5% coverage throughout the starvation periods. As an indicator of digestion in these tissues, this pattern of lysosomal activity suggests a constant digestive process within these animals. This trend does not align with the trend seen in *El*. *timida* adults [46], where the lysosomal activity is almost non-existent at the beginning of the starvation period and rises exponentially midway through the starvation period. The 25 DPM juveniles also show this pattern, starting at 0% and increasing to 8% at the end. While this is not the sharp increase seen in the adult populations, the trend is the same, starting at 0%, which indicates suppressed digestion and later rising. This further suggests that 25 DPM juveniles behave more like adults than younger juveniles concerning digestion during a starvation period. There was no discernable trend that correlated the number of functional kleptoplasts within an individual with the rate of lysosomal activity in that individual.

Juveniles that were re-introduced to food and allowed to feed for 2 hours before their lysosomal activity and kleptoplast abundance were measured showed the increased kleptoplast abundance within their digestive glands. This was not observed in adult specimens, likely because the number of kleptoplasts gained by feeding is a very small percentage compared to the number they already possess [46]. In each juvenile age group however, the increase in kleptoplast abundance is visible, accounting for a 1–15% increase in the coverage area. Also unlike the adults, every population of juveniles was observed feeding throughout the starvation period, whenever food was supplied again, suggesting they do not lose the ability to feed. The amount of kleptoplasts gained was not always significant, however. A change in lysosomal activity was never significant amongst these populations, so the digestive trends reported here are not affected by a starving slug’s recent feeding, at least within the short period between feeding and analysis.

### Comparison to short-term and non-retaining species

To collaborate and compare *El*. *timida* juvenile longevities to various long-, short- and non-retention forms, 10 other sacoglossan species, with varying abilities to perform functional kleptoplasty were starved to uncover the maximum starvation time under laboratory conditions. The non-retention forms *Placida dendritica*, *Elysia marcusi*, *Elysia tuca*, were starved, showing various longevities within the expected ranges for these particular species [48]. *Ercolania fuscata* and *Ercolania viridis* were not previously investigated in regards to maximum starvation but their starvation longevity and photosynthetic yield values allow their assignment to the Non-Retaining (NR) group, as defined by Händeler et al. [5]. Short-term retention species *Bosellia mimetica*, *Thuridilla hopei*, *Elysia papillosa*, *El*. *patina*, *El*. *cornigera* also fell within previously published ranges [4,48,55]. When compared to *El*. *timida* juveniles, these other species provide a basis for the assertion that juvenile *El*. *timida* under 15-days-old function like non-retention forms, directly digesting chloroplasts and having short lifespans in starvation.

Slugs older than 15 days begin to function like adults, having developed kleptoplast retention and a somewhat extended starvation capacity, despite lacking adult morphology and sexual maturity. A transitional stage as described by Pelletreau et al. [42] would have to begin between 15 and 25 DPM, and the intervals sampled here could not accurately define its beginning. Despite this transitional stage not appearing clearly in the data presented here, the 25 DPM juveniles are likely in this stage. They can only survive up to 22 days of starvation, a mere fifth of the time an adult *El*. *timida*. This coupled with the fact that some of these juveniles died with kleptoplasts still in their digestive glands and this has not been observed in starving adults, also suggests that these juveniles have developed many adult-like traits, but are not completely adults regarding the development of functional kleptoplasty. “Why” and “how” these animals transition from non-kleptoplast retaining individuals to long-term retaining adults remains to be resolved.

### *Elysia timida* ecology

Contrasting Marín and Ros (1992), we found adult *El*. *timida* throughout the summer months at a variety of sampling locations in Italy and Spain, however only those animals collected during May produced egg masses on a regular basis. *A*. *acetabulum* was found forming caps in May 2015 with full caps present by July 2015. This was observed again in July 2016, suggesting that the yearly cycle *A*. *acetabulum* follows is less rigid calendar than Ros and Marín (1992, 1993) reported and likely depends on locality and/or specific environmental cues that fluctuate in the natural environment. By August 2015 and 2016, no living *A*. *acetabulum* was found, only the calcified stalks from the previous season, indicating that the caps had already broken open and released their planktonic cysts. Since only the adult animals we collected in May produced egg masses, the larvae that hatched had Type 2 development [38] and they hatched when *A*. *acetabulum* is abundantly found and not completely calcified, we can confirm Marín and Ros’ (1992) observations regarding these questions.

Our experiment clearly show that functional kleptoplasty is not developed in *El*. *timida* until they are at least 15 days post-metamorphosis, and even 25 DPM individuals do not survive starvation to the degree with which adults do, but rather exhibit transient kleptoplasty. Future experiments should consider these observations when trying to understand how this remarkable ability functions and has evolved.

## Supporting information

S1 Table *Elysia timida* juvenile longevity (E_tim_juv_longevity) (Experiment 1) This workbook contains the original data points recorded during the longevity experiments. (XLSX)

S2 Table Chloroplast abundance in *Elysia timida* juveniles (E_tim_juv_cp_degradation) (Experiment 2) This workbook contains all of the raw data points exported from 3D-AMP. (XLSX)

S3 Table Digestive activity in juvenile *Elysia timida*, both starved and starved then re-introduced to food (E_tim_juv_dig_act&refeed) (Experiments 3 & 4). (XLSX)

S4 Table PAM and Longevity for all other sacoglossan species surveyed (Saco_specs_max_starvation). (XLSX)
